# How the Adequate Choice of Plant Species Favors the Restoration Process in Areas Susceptible to Extreme Frost Events

**DOI:** 10.3390/biology12111369

**Published:** 2023-10-26

**Authors:** Emerson Viveiros, Bruno Santos Francisco, Felipe Bueno Dutra, Lindomar Alves de Souza, Mariane Cristina Inocente, Aline Cipriano Valentim Bastos, Glória Fabiani Leão da Costa, Maycon Cristiano Barbosa, Rafael Paranhos Martins, Raquel Aparecida Passaretti, Maria José Pereira Fernandes, Julia Siqueira Tagliaferro de Oliveira, Ana Paula Ponce Shiguehara, Enzo Coletti Manzoli, Bruna Santos Teração, Ivonir Piotrowski, Fátima Conceição Márquez Piña-Rodrigues, José Mauro Santana da Silva

**Affiliations:** 1 Postgraduate Program in Planning and Use of Renewable Resources, Department of Environmental Sciences, Federal University of São Carlos, Campus Sorocaba, São Paulo 18052-780, Brazil; emerson.viveiros@aes.com (E.V.); fbdutra@estudante.ufscar.br (F.B.D.); lindomarsouza@estudante.ufscar.br (L.A.d.S.); mariane.inocente@estudante.ufscar.br (M.C.I.); anapps@estudante.ufscar.br (A.P.P.S.); enzomanzoli@gmail.com (E.C.M.); josemauro@ufscar.br (J.M.S.d.S.); 2 AES Brasil, Bauru 17064-868, Brazil; rafael.paranhos@aes.com (R.P.M.); raquel.passaretti@aes.com (R.A.P.); maria.fernandes@aes.com (M.J.P.F.); 3 Undergraduate Program in Forest Engineering, Department of Environmental Sciences, Federal University of São Carlos, Campus Sorocaba, São Paulo 18052-780, Brazil; mbarbosa@estudante.ufscar.br; 4 Undergraduate Program in Biologycal Sciences, Department of Biologycal Sciences, Federal University of São Carlos, Campus Sorocaba, São Paulo 18052-780, Brazil; juliasto@estudante.ufscar.br; 5 Department of Environmental Sciences, Federal University of São Carlos, Campus Sorocaba, São Paulo 18052-780, Brazil; bruna.teracao@gmail.com (B.S.T.); ivonir@ufscar.br (I.P.)

**Keywords:** climate change, ecology, morphology, seasonal forest, seedlings

## Abstract

**Simple Summary:**

Climate change is putting pressure on many researchers to adopt measures that solve the problems caused. And these problems go beyond the increase in global temperature, which, consequently, is causing an imbalance in the various natural ecosystems that remain. One of the main alternatives to combat the effects of climate change is the recovery of natural areas, be they forests, pastures, wetlands, or mountains. For that, in addition to factors such as soil preparation and post-planting management, the selection of species is fundamental to guarantee the success of the restoration. However, with the ongoing environmental imbalance, several extreme events are occurring unexpectedly, which puts pressure on the survival of some species. Frost is one of these events, which occurs when minimum temperatures are recorded in certain regions, causing various types of damage to species. Thus, the objective of this work was to evaluate how this extreme event—frost—can modify the community based on the intrinsic characteristics of some species in an area under restoration and to identify which species can resist these extreme events.

**Abstract:**

This work aimed to evaluate the impacts caused by extreme frost events in an ecological restoration area. We grouped the species in three ways: (1) type of trichome coverage; (2) shape of the seedling crown; and (3) functional groups according to the degree of damage caused by frost. The variables of the restored area and species characteristics were selected to be subjected to linear generalization analysis models (GLMs). A total of 104 individuals from seven species were sampled. The most affected species were *Guazuma ulmifolia* Lam. (98% of leaves affected), followed by *Cecropia pachystachia* Trécul and *Hymenea courbaril* L. (both 97%), *Inga vera* Willd. (84%), and *Senegalia polyphylla* (DC.) Britton & Rose with 75%. *Tapirira guianensis* Aubl. was considered an intermediate species, with 62% of the crown affected. Only *Solanum granulosoleprosum* Dunal was classified as slightly affected, with only 1.5% of leaves affected. With the GLM analysis, it was verified that the interaction between the variables of leaf thickness (Χ² = 37.1, df = 1, *p* < 0.001), trichome coverage (Χ² = 650.5, df = 2, *p* < 0.001), and leaf structure culture (Χ² = 54.0, df = 2, *p* < 0.001) resulted in a model with high predictive power (AIC = 927,244, BIC = 940,735, Χ² = 6947, R² = 0.74, *p* < 0.001). Frost-affected crown cover was best explained by the interaction between the three functional attributes (74%). We found that there is a tendency for thicker leaves completely covered in trichomes to be less affected by the impact of frost and that the coverage of the affected crown was greatly influenced by the coverage of trichomes. Seedlings with leaves completely covered in trichomes, thicker leaves, and a funneled or more open crown structure are those that are most likely to resist frost events. The success of ecological restoration in areas susceptible to extreme events such as frost can be predicted based on the functional attributes of the chosen species. This can contribute to a better selection of species to be used to restore degraded areas.

## 1. Introduction

Cold stress, which includes chilling (0–20 °C) and freezing (<0 °C) temperatures, is detrimental to plant growth and development, limiting the geographic distribution of species. This type of stress prevents the full expression of the genetic potential of plants because, in addition to directly inhibiting metabolic reactions, it causes other stresses, such as osmotic and oxidative [1]. Both temperate and tropical zone plant species can face cold stress, so the difference between the time and duration of the cold (diurnal or annual cycle) for each zone implies the strategies adopted in the different environments [2].

Under natural conditions, plants have developed mechanisms to survive cold stress. Chilling tolerance is the ability of plants to withstand low temperatures (0–15 °C) without injury or damage. Cold acclimation is an increased tolerance to the damage of freezing stress, which enables plants to survive sub-zero temperatures (<0 °C) and is acquired after previous exposure to low non-freezing temperatures [2,3].

In general, morphological symptoms of cold stress include chlorosis, curling and wilting of the leaves, tissue damage, necrosis, stunted seedlings, and growth inhibition, among others. Specifically, injuries caused by chilling stress include damage to cell membranes, alteration of membrane fluidity and lipid composition, and reduction of water potential and ATP supply. Freezing stress leads to protoplast shrinkage, cell deformation, and death [4]. It should be noted that chilling damage is a direct effect of temperature while freezing damage is the result of osmotic dehydration due to the diffusion of intracellular water into the formation of extracellular ice crystals [5].

Frost occurrence and drought are some of the main stress factors limiting the growth and geographical distribution of tree species [6]. Frost is a meteorological phenomenon that occurs when the air temperature is below 0 °C (freezing temperature), forming ice crystals on the leaves from the water vapor present in the air without transitioning to the liquid phase [7]. As a result of the stress, there is severe dehydration of the cells and freezing of the sap, leading to the rupture of plant vessels and the destruction of tissues. The level of damage the plant will suffer is influenced by the duration of exposure to the low temperature and the freezing and thawing rates [8].

Morphological and physiological differences between species can influence tolerance to the formation of ice crystals [8]. A morphological characteristic related to tolerance to water stress, but little mentioned in studies of cold stress, is the presence of trichomes on the leaves. However, Agrawal et al. (2004) have already shown that there is a correlation between leaf trichome density and frost resistance, suggesting that trichomes also play a protective role in this case [9]. Another characteristic is leaf thickness. Nautiyal et al. (2007), studying species of the genus Arachis (Fabaceae), found a positive correlation, indicating that the thicker the leaf, the lower the injury or higher the stress tolerance [10].

In South America, specifically in Brazil, frosts are frequent at latitudes higher than 19° S. At latitudes between 23° S and 27° S, frosts occur only in the winter, while at latitudes above 27° S, early frosts can occur in the fall, normal frosts in the winter, and late frosts in the spring [11]. As a result of frosts, losses have already been recorded in the country for rice, soybean, and wheat crops [12]. In addition, changes in populations and the community structure of Cerrado plant species have been reported [13,14], as have analyses of the ecophysiological responses of tree species after frosts [15]. However, there are few studies on the effect of frost in the Atlantic Forest [6], especially in restoration planting areas, despite the intense fragmentation and degradation of this biome [16].

Considering the success of forest restoration projects and future global climate conditions, it is essential to know species’ responses to different disturbances, making their selection more accurate and avoiding both species mortality and economic losses. Therefore, it is necessary to recognize the impact of extreme climatic events on restoration areas, especially in the early stages of plant development. The aim of this study was to assess how the impacts caused by extreme frost events in an ecological restoration area can shape the community structure based on the functional attributes of the planted species.

## 2. Materials and Methods

### 2.1. Geographical Location

**Figure 1:**
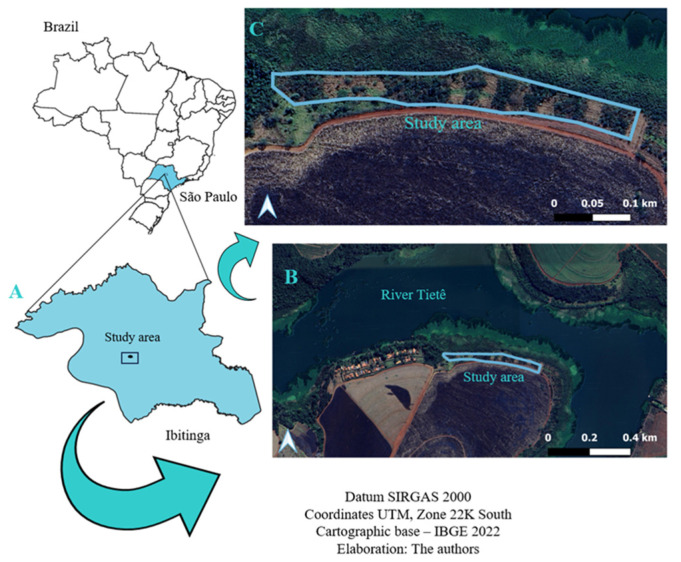
Coordinates of the studied area. (**A**) Municipality of Ibitinga-SP. (**B**) Path where the study area is located. (**C**) Study area.

The research was carried out in a nine-month-old restoration area in Ibitinga/SP, between altitudes 384 and 387 m, Latitude 21°43’ S/Longitude 49° 3’ O (Figure 1). The research area is located on a site expropriated from the Promissão Hydroelectric Power Plant reservoir on the banks of the Jacaré-Guaçu River, which for many years was used to plant sugar cane (Saccharum spp.). The region is characterized by a predominance of seasonal forest fragments in a sugar cane matrix. Ibitinga’s climate is tropical, with little rain in winter, classified as Aw by Köeppen [17]. The average temperature is 22.2 °C, and the average annual rainfall is 1231 mm. August is the driest month, with 19 mm of precipitation. The month with the highest occurrence is January, with an average of 234 mm. The warmest month of the year is January, with an average temperature of 24.8 °C. The average temperature in June is 18.5 °C [18]. The city of Ibitinga lies in the domain of the Adamantina Formation, with an Argisol-type soil [19]. The Adamantina Formation is one of the geological groups of the Western Paulista Plateau, which is part of the Paraná Sedimentary Basin [20,21].

### 2.2. Sampling

**Figure 2:**
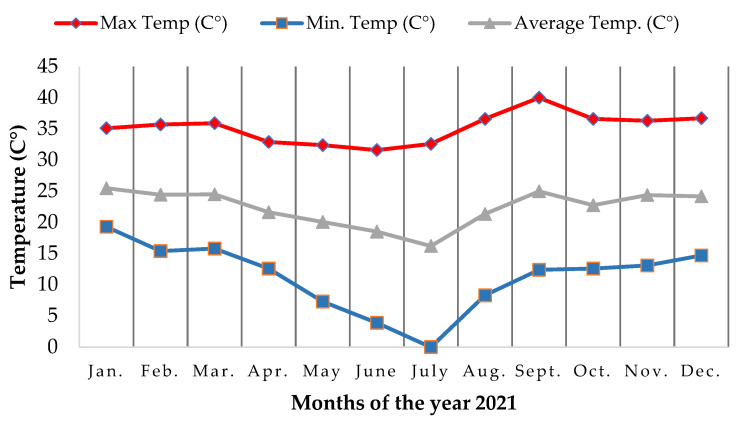
Maximum, minimum, and average temperature data for the city of Ibitinga, a city in the study area. Data were collected from the National Institute of Meteorology of Brazil [24].

The frost event occurred in July 2021 [22], reaching a minimum temperature of 0 °C (Figure 2). In August 2021, one week after the extreme frost event in the experimental area, all the planted individuals along the path were sampled, from the first planting line to the end of the area, using the zigzag walking method [23]. It estimated the height and percentage of leaves affected by frost, measured the distance from the planted seedling to the river, and counted the number of regrowth and the position of the regrowth of all the individuals sampled.

The species sampled in the field were *Tapirira guianensis* Aubl., *Inga vera* Willd., *Hymenaea courbaril* L., *Senegalia polyphylla* (DC.) Britton & Rose, *Guazuma ulmifolia* Lam., *Solanum granulosoleprosum* Dunal, and *Cecropia pachystachya* Trécul. The spelling of the scientific names, the valid names, synonyms, and the abbreviations of the authors were checked according to the Flora do Brasil [25].

**Figure 3:**
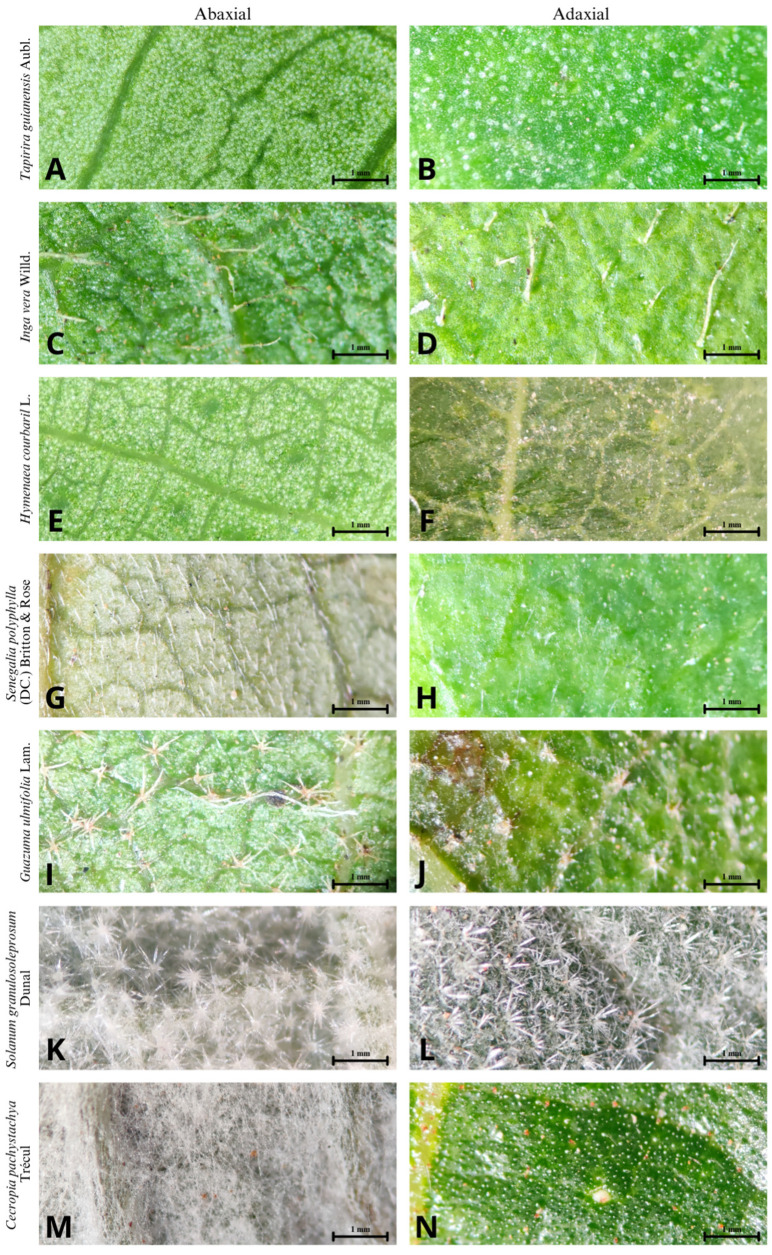
Abaxial and adaxial surfaces of the species evaluated in this study. The scale represents one millimeter. (**A**,**B**) No trichomes; (**C**,**D**) simple, claviform, glandular trichomes, scattered along the leaf blade, sometimes grouped between veins; (**E**,**F**) simple, glandular trichomes, sparse or grouped near the base of the leaflet; (**G**,**H**) no trichomes; (**I**,**J**) stellate tector trichomes, scattered along the leaf blade; (**K**,**L**) stellate trichomes, side by side, forming a layer along the entire length of the leaf blade; (**M**,**N**) simple, strigose, and uncinate trichomes, side by side, clustered across the entire abaxial leaf surface.

We collected branches with healthy, fully expanded leaves from all the species found in the sample. The leaves were taken to the laboratory for analysis of functional characteristics, such as the type and coverage of trichomes on the leaf blade and the thickness of the leaf blade. The species were grouped into three classes according to the type of trichome coverage (leaves without trichomes, leaves partially covered by trichomes, and leaves with full trichome coverage on both sides) since leaf trichomes can perform various leaf protection functions [26] (Figure 3).

**Figure 4:**
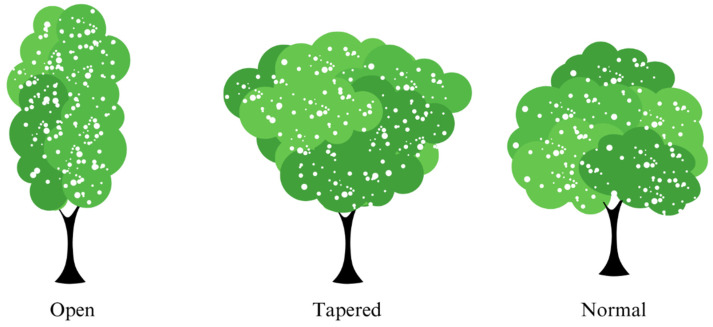
Classes of crown structures were used in this study. Tapered: the base of the crown forms an angle of less than 90°; open: the leaves emerge directly from the stem with no or short branches; normal crown: branches where the leaves come out of the branches, and the base does not form an angle of less than 90°.

The species were grouped according to the shape of the crown of seedlings in the field based on Lima [27], into three classes according to the shape of the crown (Figure 4), and the species and individuals into functional groups according to the degree of frost damage, based on Brando & Durigan [13] and Hofmann et al. [14]. It was categorized as little affected when the percentage of leaves and branches impacted was less than 34%, intermediate when the percentage was between 34% and 66%, and really affected when the impact was greater than 66%.

### 2.3. Data Analysis

To investigate the functional attributes that influence frost tolerance, mean values for the variables were obtained: percentage of trichomes, percentage of trichomes on the abaxial face, percentage of trichomes on the adaxial face, and leaf thickness for each species.

A correlation analysis between the predictor variables (height, percentage of total trichomes, percentage of trichomes on the abaxial surface, percentage of trichomes on the adaxial surface and leaf thickness, trichome coverage, leaf type, crown structure, distance from the river) and the response variable (affected crown cover) was carried out using a correlation matrix to detect redundancy between variables and, thus, eliminate false correlations.

In deciding which variable to keep, we relied on existing knowledge about the effects of frost on plant communities. Based on these analyses, the predictor variables were selected to be subjected to generalized linear model (GLM) analysis to elucidate the functional attributes that explain species’ tolerance to the effect of frost.

Different generalized linear models (GLMs) were tested to represent the relationships between these variables, considering that the best model would be the one that resulted in the highest value for the coefficient of determination (R²).

For the affected crown cover, a GLM analysis was performed with non-autocorrelated predictive variables (trichome coverage, crown structure, and leaf thickness). Thus, only variables with a correlation lower than r² = 0.7 were considered in the models in order to eliminate multicollinearity problems.

Different models were tested to represent the relationships between these variables, considering the variables in isolation and the interaction between them. We used the results of the GLM analysis to generate a biplot graph with a principal component analysis. The “Tidyverse” and “Stats” packages were used to derive the principal component analysis. Then, data-based visualizations were visualized on the first two principal components using the “Factoextra” and “Factoshiny” packages [28]. Every statistical analysis was performed using the JAMOVI 2.3.28 software [29] and in an RStudio environment [30].

## 3. Results

A total of 104 individuals were sampled from seven species distributed in seven genera and five families (Table 1). The most affected species were *Guazuma ulmifolia,* with 98% of the leaves affected, followed by *Cecropia pachystachia* and *Hymenea courbaril,* both with 97%, *Inga vera* with an average of 84%, and *Senegalia polyphylla* with 75%. *Tapirira guianensis* was considered an intermediate species, with 62% of the crown affected. Only *Solanum granulosoleprosum* was classified as slightly affected, with only 1.5% of the leaves affected.

**Figure 5:**
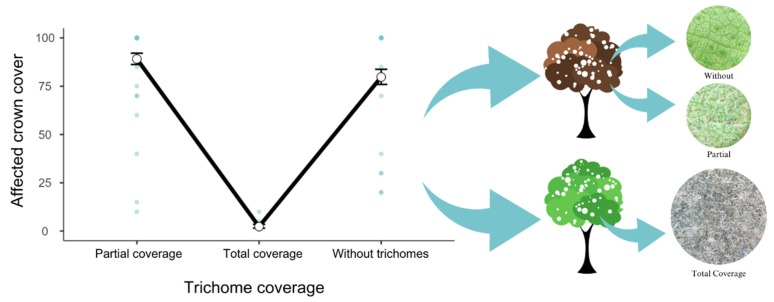
Plot resulting from the model obtained for the dependent variable affected crown cover plot from the independent variable trichome coverage (Χ² = 1399, df = 2, *p* < 0.001). In this model, it is possible to see that the percentage of the crown affected by frost is lower when the leaves have full trichome coverage.

**Figure 6:**
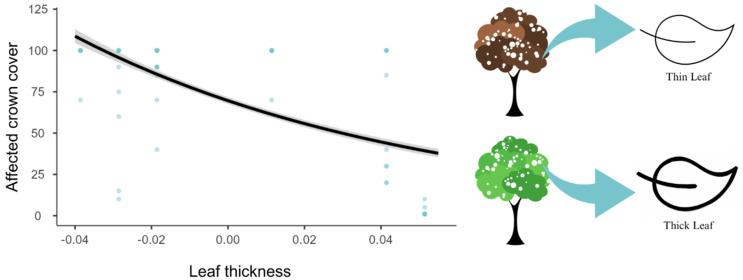
Plot resulting from the model obtained for the dependent variable affected crown cover plot from the independent variable leaf thickness (Χ² = 584, df = 1, *p* < 0.001). In this model, it is possible to observe that the percentage of the crown affected by frost decreases with leaf thickness.

**Figure 7:**
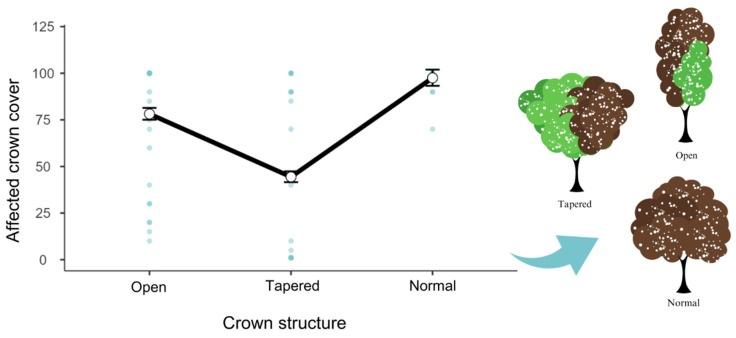
Plot resulting from the model obtained for the dependent variable affected crown cover plot from the independent variable crown structure (Χ² = 419, df = 2, *p* < 0.001). In this model, it is possible to observe that the percentage of the crown affected by frost is smaller in open and tapered crowns.

The generalized linear models (GLMs) drawn up with all the species sampled showed that 69% of the variation in the percentage of frost tolerance is explained by trichome coverage (Χ² = 1399, R² = 0.693, df = 2, *p* < 0.001) (Figure 5). Leaf thickness (Χ² = 584, R² = 0.289, df = 2, *p* < 0.001) alone explained 28% of the variation (Figure 6), and crown structure (Χ² = 419, R² = 0.208, df = 2, *p* < 0.001) explained only 20% of the variation in affected crown cover by the frost (Figure 7).

**Figure 8:**
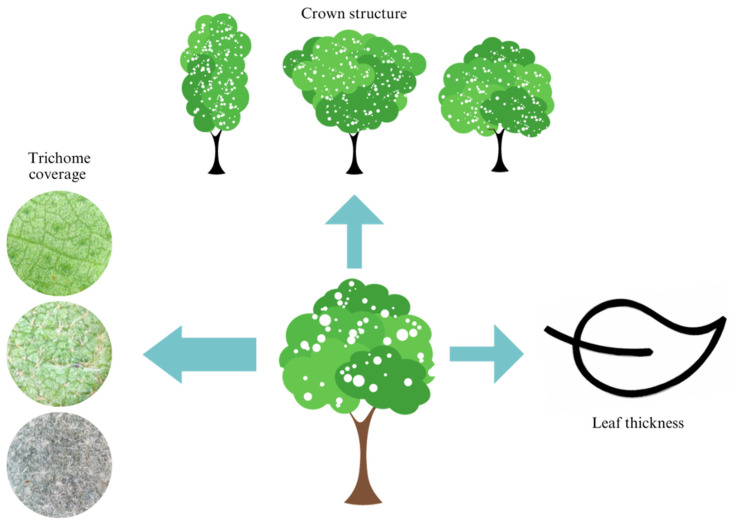
Set of functional attributes responsible for the percentage of crown affected. Trichome coverage, crown structure, and leaf thickness explained 74% of the variation in results.

The GLM analysis showed that the interaction between the three variables, was leaf thickness (Χ² = 37.1, df = 1, *p* < 0.001), trichome coverage (Χ² = 650.5, df = 2, *p* < 0. 001), and crown structure (Χ² = 54.0, df = 2, *p* < 0.001), which resulted in a model with high predictive power (AIC = 927.244, BIC = 940.735, Χ² = 6.947, R² = 0.74, *p* < 0.001). In other words, the crown cover affected was best explained by the interaction between the three functional attributes, which together explained 74% of the variation in the percentage of crown affected by the frost event (Figure 8).

**Figure 9:**
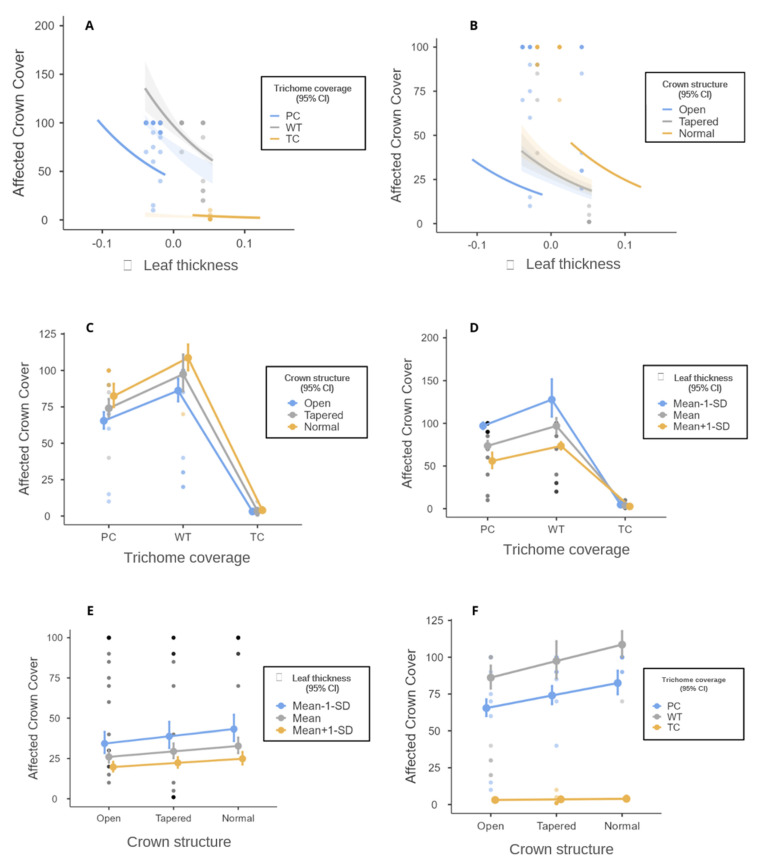
(**A**,**B**) Plots resulting from the model obtained for the dependent variable crown cover affected by the independent variables of leaf thickness on the horizontal axis, trichome coverage, and crown structure in separate lines; (**C**,**D**) plots resulting from the model obtained for the dependent variable crown cover affected by the independent variables of trichome coverage on the horizontal axis and crown structure and leaf thickness in separate lines; (**E**,**F**) plots resulting from the model obtained for the dependent variable crown cover affected by the independent variables of crown structure on the horizontal axis and leaf thickness and trichome coverage in separate lines.

It was verified that there is a tendency for thicker leaves completely covered by trichomes to be less affected by the impact of frost. Our results showed that the effects of frost on canopies with leaves not covered by trichomes or partially covered are reduced when leaf thickness is greater (Figure 9A,B).

We found that the affected crown cover was greatly influenced by the trichome coverage. When the trichome coverage is complete on the leaves, they present a high tolerance to frost (Figure 9C). However, thicker leaves are more frost-tolerant than thinner leaves, even if they lack or are partially covered by trichomes (Figure 9D).

The open crown structure characteristic showed a lower percentage of the crown affected by frost when the leaves were thicker or fully covered in trichomes (Figure 9E). Leaves with less thickness tend to suffer more from the effects of frost, especially if the crown structure is normal (Figure 9F).

**Figure 10:**
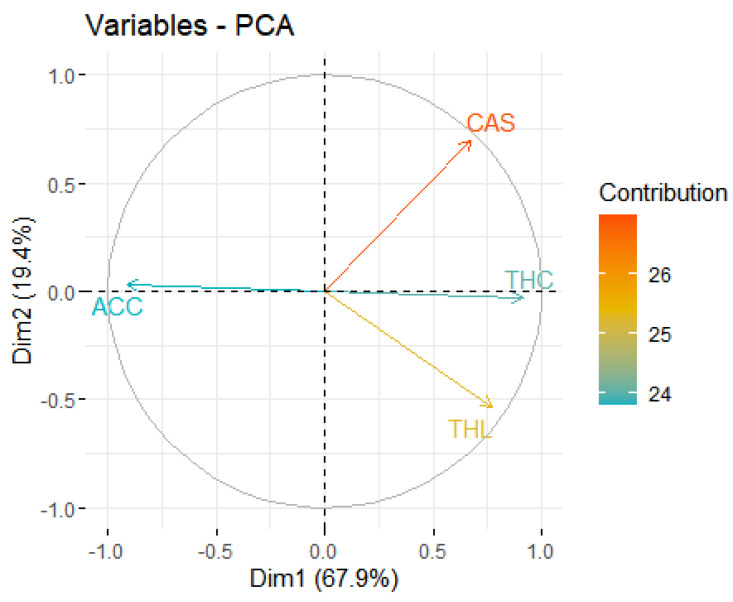
Principal component analysis (PCA) applied to the variables that best explained the GLM analysis. (A) PC1 and PC2 accounted for 67.9% and 19.4% of the total variation, respectively. ACC = affected crown cover; CAS = crown structure; THC = trichome coverage; THL = leaf thickness.

The principal component analysis (PCA) results separate the response variable and the predictor variables, with the affected crown cover being the opposite of trichome coverage, leaf thickness, and crown structure (Figure 10).

## 4. Discussion

All studied species showed injuries from the effects of frost above 64% in their canopies, except *Tapirira guianensis* and *Solanum granulosoleprosum*, which showed, respectively, 62% and 1.5% of the impact. Similar results have already been described by Durigan et al. [31] in their work “The shrub-tree flora of the middle Paranapanema”, which discusses the frost tolerance of some species, including *T. guianensis*, described as a species susceptible to frost. This work does not provide data on *S. granulosoleprosum*, but it does mention another species from the same genus, *Solanum argenteum* Dunal, which was considered a frost-tolerant species. These two species have thicker leaves, but *T. guianensis* has no trichomes, while *S. granulosoleprosum* has a leaf completely covered in stellate trichomes.

The presence of leaf trichomes can bring several benefits to these plants, depending on environmental conditions, including reducing transpiration, maintaining humidity and temperature on the leaf surface, reflecting sunlight, absorbing water, drought tolerance, and metal detoxification [26,32,33,34]. From our work, one can also add the effect of frost tolerance to these protective functions of trichomes.

Total leaf trichome coverage had a positive influence on leaf frost tolerance, explaining 69% of this variation on its own. Leaf frost tolerance was also explained by leaf thickness and crown structure, but these showed less predictive power of the frost tolerance effect (28% and 20%, respectively). The importance of trichomes has been verified in several studies that seek to explain how thicker layers of these structures can provide greater protection against ultraviolet radiation and reduce water loss in regions where drought is more pronounced and there are high temperatures [35,36,37,38,39].

Trichomes may be associated with phenolic compounds that increase the protective capacity for abiotic factors due to their diffuse deposition in cell walls. Trichomes can provide protection against UV-B radiation by acting as optical filters, filtering out wavelengths that could damage sensitive tissues [40,41]. In addition, mixtures of trichome phenolics represent a surface chemical barrier that provides protection against biotic factors (herbivory, pathogens) [42]. Thus, it was found that species with full trichome coverage have advantages in restoration areas.

However, especially for frost-affected crown cover, which is an extremely important variable in assessing the impact of frost, 31% of the data variation was not explained by total trichome coverage alone. None of the other attributes analyzed by themselves explained this variation. However, the analysis based on the interaction of the attributes was surprising. The interaction between the three attributes (trichome coverage, leaf thickness, and crown structure) resulted in high predictive power, explaining 74% of the variation in crown coverage affected by frost in the planting seedlings.

The PCA analysis (Figure 10) grouped the variables analyzed by species. The species that suffered the greatest impact from frost—*Guazuma ulmifolia*, *Cecropia pachystachia*, and *Hymenea courbaril*—were most associated with the indicator ACC (affected crown cover) and were opposite to the indicator THC (trichome coverage). This indicator (THC), in turn, was mainly associated with *Solanum granulosoleprosum*, as this species showed total trichome coverage (Figure 2), and this species was the opposite of ACC, as it was the species that suffered the least frost damage (Table 1). The *Tapirira guianensis* species was more associated with the THL (leaf thickness) indicator, as it has thicker leaves.

Our result is understandable, given the peculiar characteristics of trichomes related to protection from abiotic environmental factors (stress, water loss, drought, temperature, radiation) and biotic (herbivory, fungi, bacteria) [41], important filters that should be considered when choosing species for restoration plantations.

Species from environments that suffer from unfavorable periods, such as Tundra and Mediterranean regions, tend to have adaptive characteristics for survival, such as trichomes, thick leaves, and needles [43,44,45].

Choosing species with the greatest chance of success to be used in restoration areas, therefore, depends on deepening our knowledge of the effects of extreme events and trichome protection capacity, along with characteristics such as crown structure and leaf thickness, for as many species as possible, as this information is not yet available. Although our study was carried out for species used in restoration areas only with tree species, it is possible that the attributes of leaf thickness and total trichomes coverage are important for other forms of life, mainly vines, which are of fundamental importance for the maintenance of biodiversity in ecosystems and mainly in ecological restoration areas in times of climate change and extreme events.

## 5. Conclusions

The success of ecological restoration in areas susceptible to extreme events such as frost can be predicted based on the functional attributes of the chosen species. Seedlings with leaves completely covered in trichomes, thicker leaves, and tapered or more open crown structures are the most likely to resist frost events. These attributes are already related to protection against other stress conditions, such as water loss, temperature maintenance, herbivory protection, and radiation protection, which are desirable in ecosystems subject to extreme conditions.

Among the species analyzed, the ones least affected by frost were *Solanum granulo-soleprosum* and *Tapirira guianensis*, so their use is highly recommended for ecological restoration projects in areas susceptible to frost. On the other hand, other species, such as *Guazuma ulmifolia*, *Cecropia pachystachia*, *Hymenea courbaril*, *Inga vera*, and *Senegalia polyphylla*, did not show good adaptation to such climatic conditions. Future research could benefit from the data presented here and look for other species with similar functional characteristics to *S. granulosoleprosum* and *T. Guianensis*, basing choices on the interaction of the indicators analyzed here. These would outline a range of species suitable for use in areas susceptible to frost events, with a view to great success in ecological restoration.

## Figures and Tables

**Table 1:** List of species used in planting, with filling and diversity groups, crown shape, trichome coverage class, and frost damage categories. Captions: O = open crown; Ta = tapered crown; N = normal; TC = trichome coverage; Nt = no trichomes; Pa = partial coverage; T = total coverage; Va = very affected; I = intermediate; La = little affected.

Family	Species	Crown Structure	Class of TC	Damage Category
Anacardiaceae	*Tapirira guianensis* Aubl.	O	Nt	I
Fabaceae	*Inga vera* Willd.	Ta	Pa	Va
Fabaceae	*Hymenaea courbaril* L.	N	Nt	Va
Fabaceae	*Senegalia polyphylla* (DC.) Britton & Rose	O	Pa	Va
Malvaceae	*Guazuma ulmifolia* Lam.	N	Pa	Va
Solanaceae	*Solanum granulosoleprosum* Dunal	Ta	T	La
Urticaceae	*Cecropia pachystachya* Trécul	O	Pa	Va

## Data Availability

Not applicable.
